# New York Academy of Medicine

**Published:** 1862-04

**Authors:** J. H. Hinton

**Affiliations:** Recording Secretary, 41 West 32d Street, New York


New York Academy of Medicine.—At a Stated Meeting of the New York Academy of Medicine, held in New York March 20, 1862, the following preamble and resolutions were presented by Dr. A. H. Stevens, and unanimously adopted:

*Whereas*, during the present unhappy war many of our professional brethren in service among the combatants have risked their lives, or gone into involuntary captivity, rather than desert their sick and wounded, and have exercised their skill alike on friend and foe ; therefore,

*Be it Resolved*, That in such conduct this Academy recognizes the true spirit which should ever animate the ministers of humanity, and in testimony whereof,

*It further Resolves*, To welcome to its sittings those who have acted under these self-sacrificing and generous impulses.

J. H. Hinton, Recording Secretary,
41 West 32d Street, New York.

